# Emotional Competence, Entrepreneurial Self-Efficacy, and Entrepreneurial Intention: A Study Based on China College Students’ Social Entrepreneurship Project

**DOI:** 10.3389/fpsyg.2020.547627

**Published:** 2020-11-17

**Authors:** Chu Chien-Chi, Bin Sun, Huanlian Yang, Muqiang Zheng, Beibei Li

**Affiliations:** ^1^Business School, Foshan University, Foshan, China; ^2^Business School, Shantou University, Shantou, China

**Keywords:** college students, college social entrepreneurs, entrepreneurial intention, emotional competences, entrepreneurial self-efficacy

## Abstract

Entrepreneurship education has a lot of research on influencing factors of entrepreneurial intention but rarely studies the influence mechanism of emotional competences on entrepreneurial intention from the perspective of social entrepreneurship. This article takes college students’ social entrepreneurs as research objects, drawing on Krueger’s model, theory of planned behavior, social cognitive theory, and triadic reciprocal determinism theory. This paper constructs a conceptual model with emotional ability, entrepreneurial self-efficacy, and entrepreneurial intention, to further study their relationship. The 312 students from China College Students’ Social Entrepreneurship Project engaged in early entrepreneurship practice, conducted a questionnaire survey and used the empirical test of the structural equation model to analyze the relationship between college students’ emotional competences, entrepreneurial self-efficacy, and entrepreneurial intention. The result show: First, social–emotional competence had a positive effect on entrepreneurial intention, and the positive effect of personal affective competence on entrepreneurial intention was not supported or only partially supported. Second, all the dimensions of entrepreneurial self-efficacy were significantly and positively correlated with entrepreneurial intention. Third, emotional competence has a significant positive impact on entrepreneurial self-efficacy. Fourth, entrepreneurial self-efficacy mediated the relationship between emotional competence and entrepreneurial intention.

## Introduction

College students have the potential for innovation and entrepreneurship. Education projects and special policies increasingly provide good infrastructure, capital, and technology support to college students to promote social entrepreneurship. While the overall entrepreneurship awareness of college students is relatively low, the proportion of the class of 2018 college graduates after 6 months of self-employment was only 2.7% (Mycos Institute, 2019). At the same time, they lack a certain degree of management, anti-risk, and competences to adapt to the environment, entrepreneurial perseverance, and hard work. The failure rate among entrepreneurs is as high as 90%. Within 3 years, more than half of all college entrepreneurs will withdraw from the entrepreneurial market. The quality of entrepreneurship education in colleges and universities in China needs to be improved. However, first, the contributions of the presupport provided by colleges and policies should be acknowledged. Next, further reviews of the purpose of entrepreneurship education should be undertaken. Also, we should emphasize greatly on the study of those entrepreneurs’ psychological cognitive mechanism and their motivation.

With regard to entrepreneurship education in college, entrepreneurial intention is the strongest predictor of entrepreneurial behavior. Therefore, identifying the factors that predict entrepreneurial intention has great practical significance. The following factors have been found to influence entrepreneurial intention: decision-making mechanisms (Feola et al., 2017), entrepreneurial differences (Fini and Toschi, 2016; Roy and Das, 2019), entrepreneurship education (Xu et al., 2016; Nowiński et al., 2017; Teixeira and Forte, 2017; Wu et al., 2018), an entrepreneurial background, and environmental factors (Altinay et al., 2012; Palmer et al., 2019). The cognitive approach to entrepreneurship posits that the subjective characteristics of entrepreneurs (e.g., cognitive characteristics) influence entrepreneurial intention (Kirzner, 1978, 1997). Therefore, several scholars have examined the influence of subjective psychological factors, such as the personality traits of entrepreneurs, on entrepreneurial intention (Popescu et al., 2016; Fellnhofer, 2018; Hu et al., 2018). However, a few studies have focused on the effect of emotional competence on entrepreneurial intention, especially from the perspective of social entrepreneurship. Even fewer studies have explored the role of entrepreneurial self-efficacy in the effect of emotional competence on entrepreneurial intention. Exploring the mechanisms that underlie the effect of emotional competence on entrepreneurial intention will contribute to increased entrepreneurial awareness among college students. Emotional intelligence has been defined as the competence needed to recognize and manage one’s own and others’ feelings. Emotional competence encompasses the skills needed to feel, understand, and effectively capitalize on the power of emotions as a source of energy, information, confidence, and creativity as well as the skills needed to influence others (Goleman, 1995, 1998). Indeed, college students are more inclined to pursue self-employment than individuals who have not received college education. Liu et al. (2019), Cui and Sun (2019), Wang (2018), and Xu and Hao (2019) have found that entrepreneurship education has a significant positive effect on entrepreneurial intention among college students. Therefore, research findings that delineate the relationship between emotional competence, entrepreneurial self-efficacy, and entrepreneurial intention among college students may inform college entrepreneurship education and related training programs. Accordingly, this study aimed to determine whether (a) participation in entrepreneurship training provided by China College Students’ Social Entrepreneurship improves the emotional competence of social entrepreneurs, (b) an improvement in emotional competence influences their entrepreneurial self-efficacy, and (c) the formation of entrepreneurial intention in college students is contingent on improvements in emotional competence and entrepreneurial self-efficacy. Further, this study also sought to examine the role of entrepreneurial self-efficacy in the effect of emotional competence on entrepreneurial intention.

**FIGURE 1:**
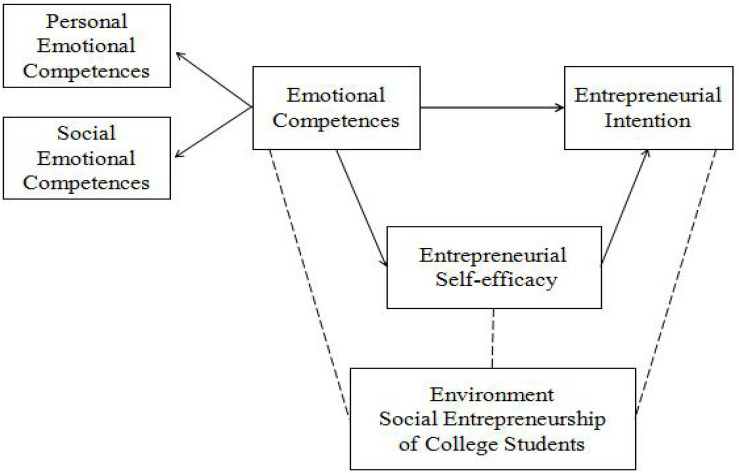
Research model.

This study was designed based on the theory of planned behavior (TPB) and social cognitive theory (SCT). According to Ajzen’s (1988,^1991^) TPB, entrepreneurial intention is influenced by attitudes, perceived behavioral control, and subjective norms. The TPB posits that the most important factor that influences behavior is intention. Behavioral intention is a necessary contributor to behavioral performance and a prerequisite for behavior (Ding and Liu, 2009). The application and practice of SCT within the domain of entrepreneurship research have revealed that entrepreneurial intention and success are largely influenced by entrepreneurial self-efficacy (Butter, 2001). The potency of entrepreneurial self-efficacy is influenced by subjective and non-subjective abilities such as entrepreneurial task difficulty, effort, and the amount of foreign aid with the environmental conditions of the tasks. The better the emotional competence of an individual is, the greater his or her awareness of entrepreneurial behavior and self-efficacy will be. Subjective competencies influence the strength of entrepreneurial self-efficacy, which is the strongest predictor of entrepreneurial intention. The basic intention-based progress model proposed by Krueger and Brazeal (1994), Yuan and Wu (2020), and Wu et al. (2020) posits that the emergence of the entrepreneurial intention process is very sensitive to initial conditions. Individuals who adopt certain behavioral goals are influenced by external factors and planned behavior attitudes. External factors include skills, knowledge, personality characteristics, and the availability of resources. Planned behavior attitudes refer to the degree of certainty of engaging in a specific behavior. Bandura and Cervone (1986) proposed the triadic reciprocal determinism theory (TRD), which posits that human behaviors are shaped by ternary interactions, are interconnected, and influence each other. Based on Krueger’s model, the TPB, SCT, and TRD, a conceptual model (Figure 1), which includes emotional competence, entrepreneurial self-efficacy, and entrepreneurial intention, was developed. The objective was to examine the relationship between these variables, offer a new perspective and basis for research on innovation and entrepreneurship in China and other countries, and provide suggestions for the long-term development of entrepreneurship education. This article is structured as follows: the section “Research Hypotheses” delineates the hypothesized relationships between emotional competence, entrepreneurial intention, and entrepreneurial self-efficacy; the section “Materials and Methods” describes the data collection procedure, the participants and sampling technique, measures used to assess the study variables, and analytic procedure; the section “Data Analysis and Results” presents the results of the analyses conducted to examine the relationship between emotional competence, entrepreneurial intention, and entrepreneurial self-efficacy; and finally, the section “Conclusion and Relevant Recommendations” are presented.

## Research Hypotheses

### Emotional Competence and Entrepreneurial Intention

Individuals with high emotional competence are better equipped to undertake entrepreneurial activities. They tend to be more creative and innovative and possess better emotional control skills. In an important situation, the interpersonal relationship will not be damaged by the emotional problems of oneself or others, which are the key factors for starting a starting a business (O’Boyle et al., 2011). Furthermore, individuals with high emotional competence demonstrate better stress tolerance, are more likely to persevere, and seek challenges. Because individuals with better emotional skills are less risk averse, they hold more positive entrepreneurial attitudes. In accordance with the TPB, Chen et al. (2018) and Yuan et al. (2019) have contended that the key to innovation and entrepreneurship education is to influence behavioral intentions related to innovation and entrepreneurship among students. Mikolajczak et al. (2007) have noted that, through self-assessments of emotional efficacy, employees can practice emotional efficacy management and improve their self-cognition competencies. This will enable them to experience greater emotional self-efficacy, take initiatives, and be creative; these changes are conducive to the formation of entrepreneurial intentions. Many studies have found that emotional competence is positively correlated with entrepreneurial intention within the domains of corporate, social, and campus entrepreneurship activities. Leutner et al. (2014) showed that differences in personality characteristics with abilities affect emotional abilities, core self-assessment, entrepreneurial trends, and attitudes are important promoters of entrepreneurial behavior. Zampetakis and Moustakis (2006) found that those with better emotional abilities are more likely to have entrepreneurial intentions. Padilla-Melendez et al. (2014) conducted a comparative analysis and found that the emotional competence of college students who participated in a training program had a significant positive effect on their entrepreneurial intentions. Bonesso et al. (2018) analyzed the emotional competences directly and the mediation effect of entrepreneurial intention, and it is concluded that higher levels of emotional competences can predict entrepreneurial intention. Further, emotional competence is reflected in personal (how individuals handle themselves) and social abilities (how individuals handle others) (Kierstead, 1999). Therefore, the following hypotheses were formulated:

Hypothesis 1: Emotional competence will have a positive effect on entrepreneurial intention among college students.

Hypothesis 1a: Personal affective competence will have a positive effect on entrepreneurial intention among college students.

Hypothesis 1b: Social–emotional competence will have a positive effect on entrepreneurial intention among college students.

### Entrepreneurial Self-Efficacy and Intention

Self-efficacy refers to one’s cognitive assessment of his or her ability to mobilize the motivation, cognitive resources, and action processes needed to control events (Wood and Bandura, 1989). According to SCT, individuals with high levels of self-efficacy demonstrate greater courage in uncertain dynamic environments. Therefore, they are more likely to be successful in achieving their personal goals. Entrepreneurial self-efficacy is a belief in entrepreneurial competences directed at itself. Thus, the higher one’s level of entrepreneurial self-efficacy is, the stronger his or her entrepreneurial intentions will be. Ding and Ding (2011) found that entrepreneurial self-efficacy has a strong predictive effect on entrepreneurial intention and the likelihood of occurrence of entrepreneurial behavior. Saulo et al. (2007) identified the following four dimensions of entrepreneurial self-efficacy: opportunity identification self-efficacy, innovation effectiveness, relationship management effectiveness, and tolerance effectiveness. Further, they found that there was a positive relationship between entrepreneurial self-efficiency and intention. Using the General Self-Efficacy Scale and Williams Creativity Tendency Scale, Tu and Wang (2017) found that there was a significant positive relationship between general self-efficacy and entrepreneurial intention among college students. Liu et al. (2018) conducted a study among college students. In accordance with SCT, entrepreneurial self-efficacy significantly and positively influenced participant ability to utilize opportunities and translate entrepreneurial intentions into behaviors. Many studies have used data from different countries and regions to examine the relationship between entrepreneurial self-efficacy and intention. Their results have consistently shown that entrepreneurial self-efficacy facilitates the formation of entrepreneurial intention (Salami, 2019). Therefore, the following hypothesis was formulated:

Hypothesis 2: Self-efficacy will have a significant positive effect on entrepreneurial intention among college students.

### Emotional Competence, Entrepreneurial Self-Efficacy, and Entrepreneurial Intention

Does emotional competence influence entrepreneurial self-efficacy and intention? According to past findings, emotional competence plays an important role in entrepreneurial intention. Individuals with high levels of self-efficacy demonstrate better stress tolerance. When problems arise, they are more likely to persevere. This nurtures their willingness to start a business (Panagiotis and Dimo, 2015). Martínez-González et al. (2019) found that subjective factors (beliefs, social norms, and values) initiate a chain of events, which influence action variables (motivation, self-efficacy, and intention). Emotional competence is also a subjective variable. Salvador (2008) examined the positive effects of emotional competence on entrepreneurial self-efficacy. Zhao et al. (2005) found that entrepreneurial self-efficacy influences the formation of entrepreneurial intention by playing an intermediary role in the relationship between personal characteristics and entrepreneurial intention. Tang and Zhang (2018) examined the direct effect of creative personality traits on entrepreneurial self-efficacy and willingness and the mediating role of entrepreneurial self-efficacy in the relationship between creative personality traits and entrepreneurial willingness. Mwiya et al. (2018) also examined the mediating role of entrepreneurial self-efficacy in the effect of different factors on entrepreneurial intention. Fernández-Pérez et al. (2019) found that entrepreneurship is facilitated by the development of emotional competence in university students. In sum, emotional abilities have a positive effect on entrepreneurial intention through entrepreneurial self-efficacy. Individuals with better emotional abilities also have better social abilities. This enables them to build and maintain relationships with potential investors (i.e., for entrepreneurship), and this promotes the formation of entrepreneurial intention. Individuals with high emotional competence are less risk averse. Therefore, they have more positive entrepreneurial attitudes, higher levels of entrepreneurial self-efficacy, and stronger entrepreneurial intentions. Therefore, the following hypotheses were formulated:

Hypothesis 3: Emotional competence will have a significant and positive effect on entrepreneurial self-efficacy among college students.

Hypothesis 4: Entrepreneurial self-efficacy will mediate the effect of emotional competence on entrepreneurial intention.

## Materials and Methods

### Sample and Questionnaire

This study used questionnaire survey method to collect data from the innovation team of universities in 19 cities in China, selected social entrepreneurship practitioners (university students) in Chinese university innovation organizations as the research objects. College Students’ Social Entrepreneurship is an international nonprofit organization that aims to promote social entrepreneurship and entrepreneurship development among college students, improve their entrepreneurial capacity, and create entrepreneurial awareness. China College Students’ Social Entrepreneurship program currently has 242 college member alliances from most parts of China, including Hong Kong. There are more than 12,000 active members. Such social entrepreneurship activities for undergraduate students promote the formation of entrepreneurial intentions and have a significant social impact. First, College Students’ Social Entrepreneurship is one of the most influential social entrepreneurship organizations. It promotes entrepreneurial practice and provides sustainability training to students. It most closely resembles real-world entrepreneurial organizations. Therefore, entrepreneurial mechanisms can be adequately examined among its members. Second, social entrepreneurship among college students is an independent development project and is not intended for general commercial entrepreneurship purposes. The formation of such entrepreneurial intentions is more universal, and scholars and educators can use relevant research findings to improve entrepreneurial education. The findings can also be used to enhance the diversity of practice methods. Third, this study was designed based on a research project undertaken by the Humanities and Social Sciences Department of Shantou University and conducted with the support of China College Students’ Social Entrepreneurship. Survey resources were provided, and the sample was heterogeneous. Therefore, the data derived from this pioneering project may better reflect the effect of emotional competence and entrepreneurial self-efficacy on entrepreneurial intention.

The questionnaire and measurement items were designed based on past findings and the characteristics of College Students’ Social Entrepreneurship. Second, the opinions of school teachers and class students were solicited. The Shantou University team test was conducted among 20 students. Based on their feedback, the questionnaire was refined. The revised questionnaire was distributed to a large number of students. Subjective variables were assessed using closed-ended items, which were rated on a five-point Likert scale. To avoid response biases, we ensured that the questionnaire did not reveal which variables were being assessed. Further, positively and negatively worded items were included. Moreover, relevant questions were included in the basic situation column to detect response bias. The survey was conducted during a national competition organized by China College Students’ Social Entrepreneurship. Team members from different universities in China responded to the questionnaire, and the samples have good timeliness and diversity. As in Table 1, the questionnaire was implemented by issuing with recycling on-site paper questionnaires. Of the 400 questionnaires that were distributed, 368 were returned, but only 312 were valid. Thus, the response rate was 92%, and the effective response rate was 78%.

**TABLE 1:** The distribution table of sample feature.

**Serial number**	**Variable**	**Category**	**Frequency**	**Percentage (%)**
1	Gender	Male	157	50.4
		Female	155	49.6
2	Profession	Humanities and social sciences	179	57.4
		Polytechnic	133	42.6
3	Roles	Participant	200	64.1
		Sponsor	112	35.9
4	Area	Developed area	254	81.4
		Less-developed area	58	18.6
Recycling questionnaire	400			
Recovery rate	368/400			92
Valid questionnaire	312			78

*1. Profession: Generally, second- and third-year college students participate in the Pioneering Bank, so there are a small number of them for more than 1 year.2. Roles: After a long period of experience in social entrepreneurship projects, they can act as initiators/innovators, playing product/service development, business plans, initiating new projects, and forming project teams. Only a few people play such roles.3. Locate first- and second-tier cities as developed regions, and third-tier cities and below as less developed regions.*

### Measures

The questionnaire used in this study was developed for use with individuals (i.e., sample units). The questionnaire consisted of three sections: demographic and other characteristics, overall improvement in the respondent with regard to the project (including the four dimensions of emotional competence and five dimensions of entrepreneurial self-efficacy), and entrepreneurial intention. The body of the questionnaire consisted of items that had to be rated on a five-point Likert scale. Participants were required to provide objective ratings for each question.

### Response Variable

Entrepreneurial intention was assessed from the perspective of the possibility of individual entrepreneurship. The items that assessed entrepreneurial intention were developed based on Krueger’ s (2000) assessment. As shown in Table 2, the factor loading of each entrepreneurial intention item was greater than 0.55. This indicates that the measurement properties of the questionnaire were acceptable. The Kaiser–Meyer–Olki statistic was 0.829, and Bartlett’s test yielded a significant value (*P* < 0.001). The cumulative variance contribution of the factors was 56.231%. These findings indicated that factor analysis could be conducted. The Cronbach’s alpha of the assessment was greater than 0.7. This indicated that the scale had adequate internal consistency.

**TABLE 2:** The reliability and validity examination of entrepreneurial intent scale.

**Variable**	**Entry**	**Cronbach alpha**	**KMO test result**	**Bartlett spherical test results**	**Factor load (varimax)**	**Explaining the amount of variation (%)**
Entrepreneurial intention	I think I am more likely to start a business in the future.	0.841	0.829	Approximate Chi-square: 745.164 df: 15 Sig: 0.000	0.81	56.231
	If I have a chance and I can make my own decision, I choose to start a business.				0.82	
	In order to be an entrepreneur in the future, I have been enriching myself.				0.68	
	After graduating, I want to start a business with people.				0.67	
	My career goal is to be an entrepreneur.				0.55	
	I am passionate about entrepreneurship.				0.59	

### Explanatory Variables

Based on the definition of emotional competence proposed by Mayer et al. (1999), emotional competence was defined as the skills needed to perceive, recognize, manage, and use one’s own or others’ emotions to tackle related problems. Accordingly, the measure of emotional competence used in this study was largely based on the revised version of the ECI-U (Goleman and Boyatzis, 2001). The assessment consisted of four sections: self-consciousness, social awareness, self-control, and social skills. The total number of items was 18 (self-consciousness and social awareness, 9 items; self-control, 4 items; and social skills, 5 items). To enhance the validity of the questionnaire, several reverse-scored items were created. As shown in Table 3, the factor loading of each item was greater than 0.5. Thus, the relevance of each question was high. Emotional competences consistency coefficient Cronbach alpha value is greater than 0.7, passed the internal consistency test.

**TABLE 3:** The reliability and validity examination of explanatory variables.

**Variable**	**Entry**	**Cronbach alpha**	**KMO test result**	**Bartlett spherical test results**	**Factor load (varimax)**	**Explaining the amount of variation (%)**
**Emotional**						
Self-awareness	Self-awareness-1	0.790	0.789	Approximate Chi-square: 343.187 df: 6 Sig: 0.000	0.70	61.448
	Self-awareness-2				0.72	
	Self-awareness-3				0.66	
	Self-awareness-4				0.71	
**Competences**						
Self-regulation	Self-regulation-1	0.785	0.830	Approximate Chi-square: 442.172 df: 15 Sig: 0.000	0.70	48.491
	Self-regulation-2				0.63	
	Self-regulation-3				0.71	
	Self-regulation-4				0.60	
**Social awareness**						
Social awareness-1	0.828	0.844	Approximate chi-square: 514.812 df: 10 Sig: 0.000	0.73	59.316	
	Social awareness-2				0.65	
	Social awareness-3				0.72	
	Social awareness-4				0.73	
	Social awareness-5				0.68	
**Social skill**						
Social skill-1	0.809	0.823	Approximate Chi-square: 537.158 df: 15 Sig: 0.000	0.58	51.427	
	Social skill-2				0.66	
	Social skill-3				0.62	
	Social skill-4				0.71	
	Social skill-5				0.71	
	Social skill-6				0.60	

### Mediating Variable

The measure of entrepreneurial self-efficacy used in this study was designed based on the scales developed by De Noble et al. (1999) and Lucas and Cooper (2004) and the contents of the self-efficacy scale developed by Schwarzer (1997). The entrepreneurial self-efficacy scale consisted of 25 items and the following five dimensions: innovation self-efficacy, risk-taking self-efficacy, opportunity identification efficacy, relationship coordination self-efficacy, and management effectiveness. To enhance the validity of the questionnaire, several reverse-scored items were created. As shown in Table 4, the factor loading of each entrepreneurial self-efficacy item was greater than 0.55. This indicated that the measurement properties of the questionnaire were good and that the relevance of each question was high. The Cronbach’s alphas of all the entrepreneurial self-efficacy dimensions were greater than 0.7. This indicated that they had adequate internal consistency.

**TABLE 4:** The reliability and validity examination of mediation variables.

**Variable**	**Entry**	**Cronbach alpha**	**KMO test result**	**Bartlett spherical test results**	**Factor load (varimax)**	**Explaining the amount of variation (%)**
**Entrepreneurial**						
Innovation self-efficacy	Innovation self-efficacy 1	0.790	0.830	Approximate Chi-square: 457.283 df: 15 Sig: 0.000	0.64	49.110
	Innovation self-efficacy 2				0.67	
	Innovation self-efficacy 3				0.67	
	Innovation self-efficacy 4				0.63	
	Innovation self-efficacy 5				0.61	
**Self-efficacy**						
Opportunity identification	Opportunity identification 1	0.809	0.861	Approximate Chi-square: 566.810 df: 21 Sig: 0.000	0.64	46.934
	Opportunity identification 2				0.62	
	Opportunity identification 3				0.65	
	Opportunity identification 4				0.60	
Risk bearing	Risk bearing 1	0.776	0.809	Approximate Chi-square: 372.283 df: 10 Sig: 0.000	0.63	52.972
	Risk bearing 2				0.55	
	Risk bearing 3				0.67	
	Risk bearing 4				0.70	
	Risk bearing 5				0.66	
Relationship self-efficacy	Relationship self-efficacy 1	0.708	0.729	Approximate Chi-square: 275.250 df: 10 Sig: 0.000	0.62	46.541
	Relationship self-efficacy 2				0.60	
	Relationship self-efficacy 3				0.61	
	Relationship self-efficacy 4				0.60	
Management self-efficacy	Management self-efficacy 1	0.866	0.906	Approximate Chi-square: 832.923 df: 21 Sig: 0.000	0.71	55.714
	Management self-efficacy 2				0.71	
	Management self-efficacy 3				0.64	
	Management self-efficacy 4				0.80	
	Management self-efficacy 5				0.69	
	Management self-efficacy 6				0.70	
	Management self-efficacy 7				0.63	

### Control Variables

According to past findings, factors such as gender, specialty, role, family background, social relationships, and the macro environment (including the economic environment, national policy, and business environment) have an impact on entrepreneurial intention. However, the scope of measurement is too broad and diffuse, and this method is vulnerable to privacy issues and response biases. Therefore, this study was conducted within the context of the China College Students’ Social Entrepreneurship project. The aforementioned variables were assessed as follows: gender, male and female; major, humanities, social sciences, and science; role, participants and initiators; and region, developed and underdeveloped. The first options were coded as “0,” and the second options were coded as “1.”

## Data Analysis and Results

### Correlation Analysis

Using the KSI matrix in LISREL 8.7, estimates for the correlations between the measurement factors were computed. The variance was set to 1 (i.e., the data were set to the standardized coefficient; Pearson’s correlation), and the factor correlation coefficient was computed using the estimation procedure. As shown in Table 5, there were significant relationships between the four dimensions of emotional competence (self-awareness, self-control, social awareness, and social skills), five dimensions of entrepreneurial self-efficacy (innovation effectiveness, opportunity recognition effectiveness, relationship effectiveness, and management effectiveness), and entrepreneurial intention. In addition, among the control variables, only gender and role were significantly correlated with entrepreneurial intention.

**TABLE 5:** Correlation analysis among latent variables.

**Variable**	**Mean**	**Standard deviation**	**Self-awareness**	**Self-regulation**	**Social awareness**	**Social skill**	**Innovation self-efficacy**	**Opportunity identification**	**Risk bearing**	**Relationship self-efficacy**	**Management self-efficacy**	**Entrepreneurial intention**
Self-awareness	4.15	0.66	1									
Self-regulation	3.87	0.63	0.504**	1								
Social awareness	4.16	0.67	0.630**	0.504**	1							
Social skill	4.08	0.61	0.672**	0.599**	0.672**	1						
Innovation self-efficacy	3.80	0.64	0.418**	0.594**	0.418**	0.550**	1					
Opportunity identification	3.86	0.60	0.546**	0.634**	0.546**	0.638**	0.665**	1				
Risk bearing	3.94	0.69	0.545**	0.573**	0.431**	0.505**	0.408**	0.519**	1			
Relationship self-efficacy	3.95	0.55	0.602**	0.633**	0.502**	0.660**	0.608**	0.654**	0.546**	1		
Management self-efficacy	4.08	0.66	0.524**	0.589**	0.524**	0.612**	0.466**	0.532**	0.574**	0.629**	1	
Entrepreneurial intention	3.71	0.76	0.228**	0.491**	0.228**	0.372**	0.416**	0.454**	0.456**	0.504**	0.300**	1

*Significant at the level of *P* < 0.05; **significant at the level of *P* < 0.01.

### Structural Equation Modeling: Emotional Competence, Entrepreneurial Self-Efficacy, and Entrepreneurial Intention

Structural equation modeling was used to test the study hypotheses. In other words, we examined the relationship between emotional competence, entrepreneurial self-efficacy, and entrepreneurial intention and the mediating role of entrepreneurial self-efficacy in the relationship between emotional competence and entrepreneurial intention. First, structural equation modeling was used to examine the relationship between emotional competence and entrepreneurial self-efficacy. As shown in Table 6, the Chi-squared statistic and degrees of freedom for Models 1, 2, and 3 were lower than the acceptable threshold of 5. The RMR and RMSEA values were lower than 0.08. The GFI, NFI, NNFI, CFI, and AGFI values reached the minimum required value of 0.90. These findings indicate that Models 1, 2, and 3 fit the data well.

**TABLE 6:** The fitting index of structural equation models M1, M2, and M3.

**Indicator name**	**Ideal value**	**M1 model**	**M2 model**	**M3 model**
P值	0.000	0.000	0.000	0.000
X/df	≤5	37.0/8 = 4.6	79.5/19 = 4.2	78.0/22 = 3.5
RMSEA	≤0.06	0.68	0.08	0.59
RMR	≤0.08	0.054	0.065	0.037
GFI	≥0.9	0.96	0.94	0.95
AGFI	≥0.8	0.90	0.89	0.88
NFI	≥0.9	0.97	0.96	0.98
NNFI	≥0.9	0.95	0.95	0.97
CFI	≥0.9	0.97	0.97	0.98

**FIGURE 2:**
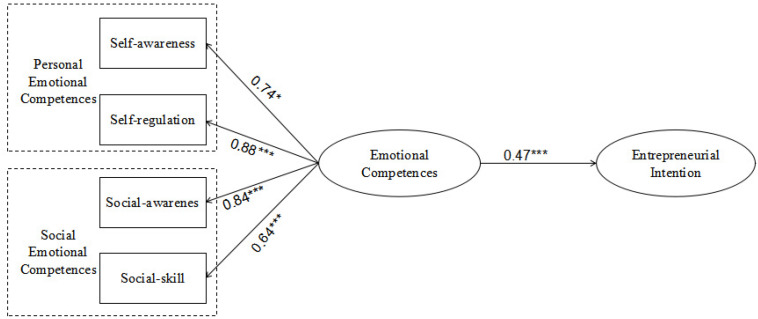
The path map M1 of emotional ability to entrepreneurial intention.

With regard to the path from emotional competence to entrepreneurial intention (Figure 2), emotional competence had a direct positive effect on entrepreneurial intention. The standardized coefficient was 0.47, and the corresponding *t*-value was 4.16 (which is substantially greater than 1.96) and significant. This indicated that emotional competence had a positive effect on entrepreneurial intention. Thus, Hypothesis 1 was supported. The standardized coefficient that emerged for self-consciousness was 0.74 (*t* < 1.96) and nonsignificant. Thus, Hypothesis 1a, which predicted that personal affective competence will have a positive effect on entrepreneurial intention, was not supported. However, as predicted by Hypothesis 1b, social-emotional competence had a positive effect on entrepreneurial intention.

**FIGURE 3:**
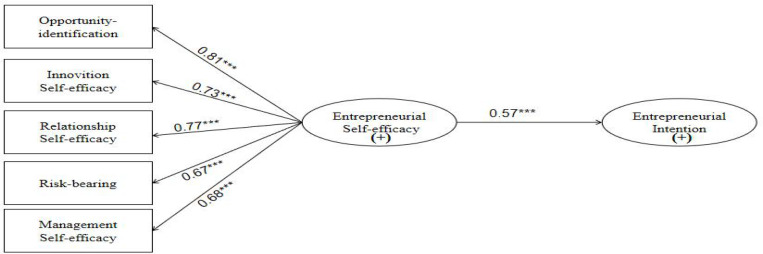
The path map M2 of entrepreneurial self-efficacy to entrepreneurial intention. ***significant at the level of *P* < 0.001.

In the path map Model 2 of entrepreneurial self-efficacy versus entrepreneurial intention in Figure 3, the entrepreneurial self-efficacy has a direct impact on entrepreneurial intention. The standardized coefficient was 0.57, and the corresponding *t*-value was 8.79 (which is much higher than 1.96) and significant. Therefore, Hypothesis 2 was supported.

**FIGURE 4:**
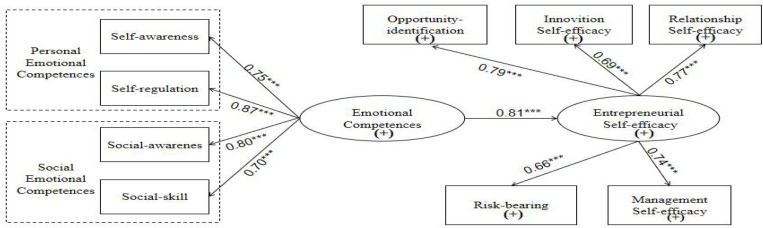
The direct model M3 of emotional competence for entrepreneurial self-efficiency. ***significant at the level of *P* < 0.001.

With regard to the path from emotional competence to entrepreneurial self-efficacy (Model 3, Figure 4), the standardized coefficient was 0.93, and the corresponding *t*-value was 12.56 (which is much higher than the required significance level; *t* = 1.96). This indicated that emotional competence had a positive effect on entrepreneurial self-efficacy. Thus, Hypothesis 3 was supported. Similarly, the five dimensions of entrepreneurial self-efficacy, namely, opportunity identification effectiveness, innovation effectiveness, relationship effectiveness, risk-taking effectiveness, and management effectiveness had factor loadings greater than 0.66, and the corresponding *t*-values were greater than the minimum required significance level (*t* = 1.96). Emotional competence had a positive effect on opportunity recognition effectiveness, innovation effectiveness, relationship effectiveness, risk tolerance effectiveness, and management effectiveness (i.e., entrepreneurial self-efficacy). Thus, Hypothesis 3 was further supported.

### The Mediating Role of Entrepreneurial Self-Efficacy

**FIGURE 5:**
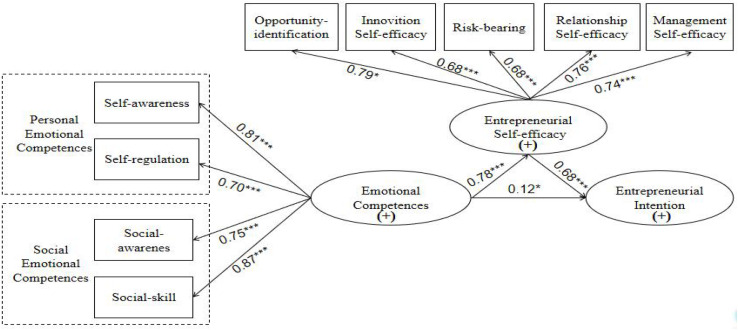
The integration model M4 of entrepreneurial self-efficacy as a mediator variable. *Significant at the level of *P* < 0.05; ***significant at the level of *P* < 0.001.

LISREL was used to analyze Model 4 and examine the role of entrepreneurial self-efficacy in the relationship between emotional competence and entrepreneurial intention (Figure 5). As shown in Table 7, the fit indices that emerged for Model 4 reached the accepted thresholds: RMSEA = 0.07 (should be less than 0.08), GFA = 0.80 (should be greater than 0.8), and RMR = 0.06 (should approximate 0.06). The NFI, NNFI, and CFI values were all greater than 0.9. Thus, Model 4 was found to be a good fit for the data.

**TABLE 7:** The data fitting results of structural equation model M4.

**Structural equation model**	**X/df**	***P*-value**	**RMSEA**	**RMR**	**GFI**	**NFI**	**NNFI**	**CFI**
M4	1,086.07/458 = 2.06	0.000	0.07	0.06	0.80	0.94	0.96	0.97

Further, we examined whether entrepreneurial self-efficacy mediated the aforementioned relationship fully or partially. As per the guidelines provided by Baron and Kenny (1986), if the path from emotional competence to entrepreneurial intention is not significant, then it can be concluded that entrepreneurial self-efficacy fully mediated the relationship. Otherwise, it can be concluded that it played a partial mediating role. As shown in Table 8, the direct effect of emotional competence on entrepreneurial self-efficacy was significant, but the indirect effect was not significant. The total effect of emotional competence on entrepreneurial intention was significant (*t* = 6.07). The direct effect was significant (*t* = 1.99), and the indirect effect was also significant. The corresponding *t*-value was 2.76 (i.e., substantially higher than 1.96). When entrepreneurial self-efficacy was included in the model, the significant positive relationship between emotional competence and entrepreneurial intention remained significant. Thus, Hypothesis 4 was supported. Entrepreneurial self-efficacy partially mediated the relationship between emotional competence and entrepreneurial intention.

**TABLE 8:** Analysis of effect of the latent variable path analysis.

**Variable**	**Entrepreneurial self-efficacy**		**Entrepreneurial intention**	
	**Normalization coefficient**	***t*-Value**	**Normalization coefficient**	***t*-Value**
**Emotional competences**				
Direct effect	0.78	14.85***	0.12	1.99*
Indirect effect	–	–	0.71	2.76***
Overall effectiveness	0.78	14.85***	0.54	6.07***
**Entrepreneurial self-efficacy**				
Direct effect	–	–	0.68	2.79***
Indirect effect	–	–	–	–
Overall effectiveness	–	–	0.68	2.79***

When the value of t is greater than 1.96, *P < 0.05; when it is greater than 2.58, **P < 0.01; when it is greater than 3.96, ***P < 0.001.

## Conclusion and Recommendations

### Conclusion

Based on the present findings, several conclusions can be drawn. First, social–emotional competence had a positive effect on entrepreneurial intention, and the positive effect of personal affective competence on entrepreneurial intention was not supported or only partially supported. When their emotional competence improves, college students feel more confident about their entrepreneurial abilities. This helps them seize entrepreneurial opportunities. Second, all the dimensions of entrepreneurial self-efficacy were significantly and positively correlated with entrepreneurial intention. In other words, the higher their level of entrepreneurial self-efficacy was, the stronger their entrepreneurial intentions were. Third, the four dimensions of emotional competence (self-awareness, self-control, social awareness, and social skills) were positively related to the five dimensions of entrepreneurial self-efficacy (innovation effectiveness, opportunity recognition effectiveness, management effectiveness, risk tolerance effectiveness, and relationship effectiveness). Therefore, emotional competence has a significant positive impact on entrepreneurial self-efficacy. Fourth, entrepreneurial self-efficacy mediated the relationship between emotional competence and entrepreneurial intention. The present findings are consistent with the results of Salvador’s (2008) study in which emotional competence had a positive effect on the self-efficacy of entrepreneurs. Further, they enrich our understanding of the results reported by Tang and Zhang (2018) and Mwiya et al. (2018). They also delineate the relationship between emotional competence, self-efficacy, and entrepreneurial intention. In sum, emotional competence reinforced entrepreneurial intention by improving entrepreneurial self-efficacy. It is important to cultivate college students’ emotional abilities, including the management of personal and social–emotional abilities, and continuously provide guidance for future entrepreneurial endeavors. This is an important and effective means by which entrepreneurial intentions can be cultivated among college students (Baron and Kenny, 1986).

### Recommendations

First, the development of emotional competence, especially the ability to overcome communication barriers, among college students should be emphasized. Entrepreneurship training programs should be provided to college students. The cultivation and application of emotional abilities will not only enhance their self-confidence and independence, help them build good interpersonal relationships, and respond to stress adaptively but also help them achieve resource integration at work or during entrepreneurial activities. Actively carrying out the activities about cultivating and applying college students’ emotional abilities in colleges, including adding related training in emotional use and management, stress response and management, interpersonal relationship coordination; providing scenario simulation, experience training, quality development, and social practice so as to improve the emotional competences of college students.

Second, drawing upon existing theories, college students should be differentiated based on their emotional competence and entrepreneurial self-efficacy levels. This will facilitate the exploration of a more comprehensive mechanism to promote innovation and nurture talent. Even though the emotional abilities of students are classified as abilities, entrepreneurial self-efficacy is a subjective assessment and belief (i.e., that one possesses the abilities needed for entrepreneurship) and is classified as a self-assessment of abilities. These two factors are closely related. Enhancing the emotional competence of college students will improve their entrepreneurial self-efficacy. Therefore, it is necessary and important to emphasize the cultivation and use of emotional abilities, underscore the role of entrepreneurial self-efficacy, actively establish personnel training measures, flexibly promote classroom diversity, nurture innovative thinking, and improve the practical abilities of students (Zeng et al., 2017).

The importance of strengthening the development of early-stage entrepreneurship practice platforms cannot be emphasized enough. This will foster collaboration between teachers and students and promote after-class practice. They serve as an important platform through which entrepreneurial intentions can be cultivated among college students. Equipped with good projects and ideas, college students will have to only take the first steps toward an entrepreneurial “long march.” Indeed, college students are afforded limited work experience. In general, when they explore business opportunities or entrepreneurial projects, they tend to remain in the theoretical analysis stage. When they are faced with problems and risks, college students tend to behave immaturely. As a result, their entrepreneurial ventures often result in failure. The development of an early entrepreneurial practice platform will ensure that students interact with their communities more closely, actively engage in practical activities, and translate their creative ideas into action. In this manner, they can explore and identify their strengths. Through collective effort, one can provide practical experience, management experience, business knowledge, resources, and assistance to cultivate thinking patterns for acquired entrepreneurship. This will help college students form more stable entrepreneurial intentions and lay the foundation for future entrepreneurial actions.

## Data Availability Statement

The original contributions presented in the study are included in the article/supplementary material, further inquiries can be directed to the corresponding author.

## Ethics Statement

Ethical review and approval was not required for the study on human participants in accordance with the local legislation and institutional requirements. Written informed consent to participate in this study was provided by the patient/participants’ or patient/participants legal guardian/next of kin.

## Author Contributions

CC-C and MZ planned the study, collected the data, and wrote the manuscript. MZ, BS, and BL analyzed the data and wrote the manuscript. Three graduate students collected the data and wrote the manuscript. All authors listed have made a substantial, direct and intellectual contribution to the work, and approved it for publication.

## Conflict of Interest

The authors declare that the research was conducted in the absence of any commercial or financial relationships that could be construed as a potential conflict of interest.

## References

[B1] Ajzen I. (1988). *Attitudes, Personality, and Behavior.* Buckingham: Open University Press.

[B2] Ajzen I. (1991). The theory of planned behavior. *Organ. Behav. Hum. Decis. Process.* 50 179–211. 10.1016/0749-5978(91)90020-t

[B3] Altinay L. Madanoglu M. Daniele R. Lashley C. (2012). The influence of family tradition and psychological traits on entrepreneurial intention. *Int. J. Hosp. Manag.* 31:499 10.1016/j.ijhm.2011.07.007

[B4] Bandura A. Cervone D. (1986). Differential engagement in self-reactiveinfluences in cognitively-based motivation. *Organ. Behav. Hum. Decis. Process.* 88 92–113. 10.1016/0749-5978(86)90028-2

[B5] Baron R. M. Kenny D. A. (1986). The moderator-mediator variable distinction in social psychological research: conceptual, strategic and statistical considerations. *J. Pers. Soc. Psychol.* 51 1173–1182. 10.1037/0022-3514.51.6.1173 3806354

[B6] Bonesso S. Gerli F. Pizzi C. Cortellazzo L. (2018). Students’ entrepreneurial intentions: the role of prior learning experiences and emotional, social, and cognitive competencies. *J. Small Bus. Manag.* 1:jsbm.12399 10.1111/jsbm.12399

[B7] Butter E. H. (2001). Examining female entrepreneurs’ management style: an application of a relational frame. *J. Bus. Ethics* 29 253–270.

[B8] Chen Y. Jia W. S. Zheng Y. J. (2018). Rational reflection and mode construction:innovation and entrepreneurship education of higher vocational colleges. *Res. High. Educ. Eng.* 2 170–175.

[B9] Cui J. Sun J. H. (2019). Research on the intermediary mechanism of college entrepreneurship education affecting college students’ entrepreneurial mind-based on the perspective of entrepreneurial emotion. *High. Educ. Manag.* 7 108–115. 10.12677/ASS.2019.85103

[B10] De Noble A. F. Jung D. I. Ehrlich S. B. (1999). *Entrepreneurial Self-Efficacy: The Development of a Measure and its Relationship to Entrepreneurial Action.* Waltham, MA: P&R Publication Inc.

[B11] Ding M. L. Ding S. W. (2011). An Empirical study of college students’ entrepreneurial self-efficacy, behavior control perception and entrepreneurial intention. *Stat. Inf. Forum.* 3 108–112. 10.1108/17561391111144573

[B12] Ding M. L. Liu B. X. (2009). Venture research: the theoretical origins and research directions from trait view to cognitive view. *Mod. Manage. Sci.* 8:124. 10.1038/embor.2009.124 19636302 PMC2726000

[B13] Fellnhofer K. (2018). Narratives boost entrepreneurial attitudes: making an entrepreneurial career attractive? *Eur. J. Educ.* 4:274. 10.1111/ejed.12274 29863170 PMC5969248

[B14] Feola R. Vesci M. Botti A. Parente R. (2017). The determinants of entrepreneurial intention of young researchers: combining the theory of planned behavior with the triple helix model. *J. Small Bus. Manag.* 57 1424–1443. 10.1111/jsbm.12361

[B15] Fernández-Pérez V. Montes-Merion A. Rodríguez-Ariza L. Alonso Galicia P. E. (2019). Emotional competencies and cognitive antecedents in shaping student’s entrepreneurial intention: the moderating role of entrepreneurship education. *Int. Entrepp. Manag J.* 15 281–305.

[B16] Fini R. Toschi L. (2016). Academic logic and corporate entrepreneurial intentions: a study of the interaction between cognitive and institutional factors in new firms. *Int. Small Bus. J.* 76 252–258. 10.1177/0266242615575760

[B17] Goleman D. (1995). *Emotional Intelligence.* New York, NY: Bantam Books.

[B18] Goleman D. (1998). *Working With Emotional intelligence.* New York, NY: Bantam Books.

[B19] Goleman D. Boyatzis R. E. (2001). *Emotional Competence Inventory.* Boston, MA: Hay Group.

[B20] Hu R. Wang L. Zhang W. Bin P. (2018). Creativity, proactive personality, and entrepreneurial intention: the role of entrepreneurial alertness. *Front. Psychol.* 9:951. 10.3389/fpsyg.2018.00951 29962985 PMC6011088

[B21] Kierstead J. (1999). Human Resource Management Trends and Issues: Emotional Intelligence (EI) in the Workplace. Research Directorate, Policy Research and Communications Branch, Public Service Commission Branch, Public Service Commission of Canada. Available online at: http://www.psc-cfp.gc.ca/research/personnel/ei_e.htm (accessed October 2019).

[B22] Kirzner I. M. (1978). *Competition and Entrepreneurship.* New York, NY: Economics Books.

[B23] Kirzner I. M. (1997). Entrepreneurial discovery and the competitive market process: an austrian approach. *J. Econ. Lit.* 35 60–85. 10.2307/2729693

[B24] Krueger N. F. (2000). The cognitive infrastructure of opportunity emergence. *Entrep. Theory Pract.* 24 5–23. 10.1177/104225870002400301

[B25] Krueger N. F. Brazeal D. V. (1994). Entrepreneurial potential and potential entrepreneurs. *Entrep. Theory Pract.* 18 91–104.

[B26] Krueger N. F. Reilly M. D. Carsrud A. L. (2000). Competing models of entrepreneurial intentions. *J. Bus. Ventur.* 15 411–432.

[B27] Leutner F. Ahmetoglu G. Akhtar R. (2014). The relationship between the entrepreneurial personality and the Big Five personality traits. *Pers. Individ. Differ.* 63 58–63. 10.1016/j.paid.2014.01.042

[B28] Liu J. W. Wu J. L. Gu J. B. (2018). Entrepreneurial self-efficacy and opportunity recognition: the moderating effect of entrepreneurship education. *Sci. Technol. Manage. Res.* 38 210–216. 10.1145/335.35079

[B29] Liu X. Y. Lin C. Zhao G. Zhao D. (2019). Research on the effects of entrepreneurial education and entrepreneurial self-efficacy on college students’ entrepreneurial intention. *Front. Psychol.* 10:869. 10.3389/fpsyg.2019.00869 31068862 PMC6491517

[B30] Lucas W. A. Cooper S. Y. (2004). Enhancing self-efficacy to enable entrepreneurship: the case of CMI’s connections. *Ssrn Electronic.* 44 489–604.

[B31] Martínez-González J. Kobylinska U. Garcia-Rodriguez F. J. Nazarko L. (2019). Antecedents of entrepreneurial intention among young people: model and regional evidence. *Sustainability* 11:6993 10.3390/su11246993

[B32] Mayer J. D. Caruso D. R. Salovey P. (1999). Emotional intelligence meets traditional standards for an intelligence. *Intelligence* 27 267–298.

[B33] Mikolajczak M. Roy E. Luminet O. (2007). The moderating impact of emotional intelligence on free cortisol responses to stress. *Psychoneuroendocrinology* 32 1000–1012. 17935898 10.1016/j.psyneuen.2007.07.009

[B34] Mwiya B. M. K. Wang Y. Kayekesi M. Bernadette K. (2018). Exploring entrepreneurial intention’s mediating role in the relationship between self-efficacy and nascent behaviour: evidence from Zambia, Africa. *J. Small Bus. Enterp Dev.* 3:83 10.1108/JSBED-03-2017-0083

[B35] Mycos Institute (2019). *Chinese 4-Year College Graduates, Employment Annual Report. Social Sciences.* Cambridge, MA: Academic Press.

[B36] Nowiński W. Haddoud M. Y. Lančarič D. Egerova D. Czegledi C. (2017). The impact of entrepreneurship education, entrepreneurial self-efficacy and gender on entrepreneurial intentions of university students in the Visegrad countries. *Stud. High. Educ.* 8 1–19.

[B37] O’Boyle E. H. Humphrey R. H. Pollack J. M. Hawver T. H. Story P. A. (2011). The relation between emotional intelligence and job performance: a meta-analysis. *J. Organ. Behav.* 32 788–818. 10.1002/job.714

[B38] Padilla-Melendez A. Fernandez-Games M. Molina-Gomez J. (2014). Feeling the risk: effects of the development of emotional competences with outdoor training on the entrepreneurial intent of university students. *Int. Entrep. Manag. J.* 10 861–884. 10.1007/s11365-014-0310-y

[B39] Palmer C. Fasbender U. Kraus S. Bikner S. (2019). A chip off the old block? - The role of dominance and parental entrepreneurship for entrepreneurial intentions. *Rev. Manag. Sci.* 5:e0342-17 10.1007/s11846-019-00342-17

[B40] Panagiotis P. Dimo D. (2015). Burst bubbles or build steam? entrepreneurship education, entrepreneurial self-efficacy, and entrepreneurial intentions. *J. Small Bus. Manag.* 53 970–985. 10.1111/jsbm.12116

[B41] Popescu C. C. Bostan L. Robu L.-B. Maxim A. (2016). An analysis of the determinants of entrepreneurial intentions among students: a Romanian case study. *Sustainability* 8 771–793. 10.3390/su8080771

[B42] Roy R. Das N. (2019). A critical comparison of factors affecting science and technology students’ entrepreneurial intention: a tale of two genders. *Int. J. Educ. Vocat. Guid* 20 49–77.

[B43] Salami S. O. (2019). Examining the emerging entrepreneurial mindset in adolescence: a study in Nigeria. *Int. J. Psychol.* 2:431. 10.1002/ijop.12431 28493413

[B44] Salvador W. R. (2008). The entrepreneurial self-efficacy of nascent entrepreneur: the case of two economics in transition. *J. Enterp. Cult.* 10 109–110. 10.1142/S021 8495802000165

[B45] Saulo D. B. Megan W. Gerhardt J. R. K. (2007). The role of cognitive style and risk preference on entrepreneurial self-efficacy and entrepreneurial intentions. *Leadersh. Organ. Stud.* 13 63–77.

[B46] Schwarzer R. (1997). The assessment of optimistic self-beliefs: comparison of the German, Spanish, and Chinese version of the general self-efficacy scale. *Appl. Psychol.* 46 69–88. 10.1111/j.1464-0597.1997.tb01096.x

[B47] Tang Y. Z. Zhang Y. X. (2018). Research on the impact of college students’ creative personality on entrepreneurial willingness–mediating effect based on entrepreneurial self-efficacy. *High. Edu. Explore* 4:791.

[B48] Teixeira A. A. C. Forte R. P. (2017). Prior education and entrepreneurial intentions: the differential impact of a wide range of fields of study. *Rev. Manag. Sci.* 11 1–42. 10.1007/s11846-015-0188-2

[B49] Tu Y. Wang H. (2017). A study on the influence of college students’ self-efficacy and creativity on entrepreneurial intention. *J. High. Educ*. 3 95–100.

[B50] Wang Z. H. (2018). A Study on the transmission mechanism of the effect of college entrepreneurship education on college students’ entrepreneurial intention. *Technol. Econ.* 8 76–80. 10.1177/2515127419860307

[B51] Wood R. Bandura A. (1989). Social cognitive theory of organizational management. *Acad. Manag. Rev.* 14 361–384. 10.2307/258173

[B52] Wu Y. J. Liu W. J. Yuan C. H. (2020). A mobile-based barrier-free service transportation platform for people with disabilities. *Comput. Hum. Behav.* 107:105776 10.1016/j.chb.2018.11.005

[B53] Wu Y. J. Yuan C. H. Pan C. I. (2018). Entrepreneurship education: an experimental study with information and communication technology. *Sustainability* 10:691.

[B54] Xu H. Hao L. (2019). Research on the impact of entrepreneurship education on entrepreneurship willingness of college students–multiple intermediary effects based on entrepreneurial self-efficacy. *Sci. Technol. Ind.* 8 103–108.

[B55] Xu X. Ni H. Ye Y. (2016). Factors influencing entrepreneurial intentions of Chinese secondary school students: an empirical study. *Asia Pac. Educ. Rev.* 17 1–11. 10.1007/s12564-016-9439-4

[B56] Yuan C. H. Wu Y. J. (2020). Mobile instant messaging or face-to-face? Group interactions in cooperative simulations. *Comput. Hum. Behav.* 113:106508 10.1016/j.chb.2020.106508

[B57] Yuan C. H. Wu Y. J. Tsai K. M. (2019). Supply chain innovation in scientific research collaboration. *Sustainability* 11:753 10.3390/su11030753

[B58] Zampetakis L. A. Moustakis V. (2006). Linking creativity with entrepreneurial intentions: a structural approach. *Int. Entrep. Manag. J.* 2 413–428.

[B59] Zeng F. Q. Qian L. Cui X. R. (2017). Can emotional competences affect the entrepreneurial intent of social entrepreneurs?——the empirical study on Enactus in China. *Innovation* 11 20–27. 10.1371/journal.pone.0090051 24594546 PMC3940715

[B60] Zhao H. Seibert S. E. Hill G. E. (2005). The mediating role of self-efficacy in the development of entrepreneurial intentions. *J. Appl. Psychol.* 90 1265–1272. 16316279 10.1037/0021-9010.90.6.1265

